# COVID-19 and ocular implications: an update

**DOI:** 10.1186/s12348-020-00212-4

**Published:** 2020-09-04

**Authors:** Raffaele Nuzzi, Luigi Ludovico Carucci, Flavia Tripoli

**Affiliations:** Clinica Oculistica Universitaria Città della Salute e della Scienza di Torino, via Cherasco 23, Torino (TO), Italy

**Keywords:** COVID-19, Conjunctivitis, Keratoconjunctivitis, Tear, Conjunctival secretion, Contact lens, Hydroxychloroquine, Guidelines

## Abstract

**Background:**

COronaVIrus Disease 19 (COVID-19) was first reported in Wuhan, China in December 2019 and is now pandemic all over the world. The purpose of this review is to highlight the possible ocular presentation of COVID-19 infection and the consequence of the pandemic in the daily ophthalmology routine. A total of 9 articles was included by searching PubMed database for articles published between December 2019 and April 2020.

**Main body:**

Conjunctivitis (and keratoconjunctivitis) can be the first symptom in infected patients. The virus can be present in tear and conjunctival secretions, requiring maximum attention. It’s important to understand if COVID-19 could spread through the ocular route or present as the primary infected site.

Ocular implications should also be considered for therapy. In fact, one potential treatment is chloroquine and its derivatives, including hydroxychloroquine. Hydroxychloroquine, in fact, can induced retinal toxicity.

The exponential increase in lthe number of Covid-19 cases was like a tsunami for health care companies, which were not ready to face this emergency. Ophthalmology departments were also affected by the reorganization of healthcare services.

**Conclusion:**

The studies analaysed have some limitations. First, the sample size and the covered population consisted mainly of patients with mild disease. Moreover, the studies are often descriptive study, without a correlation analysis.

Finally, no normal population was observed in the studies, so a normal control group should be included for comparison in future studies.

With the evolving COVID-19 pandemic and with its high infectivity, it is necessary to rearrange ophthalmologist routine clinical practice in order to control viral spread and try to maximize patient and health-care provider’s safety.

## Background

In December 2019, a number of cases with pneumonia of unexplained cause occurred in Wuhan, China. Deep sequencing from lower respiratory tract samples confirmed infection was caused by a novel coronavirus, named coronavirus disease 2019 (COVID-19) by the World Health Organization [1].

On 11th March 2020, due to the global logarithmic expansion of the cases, the coronavirus disease 2019 (COVID-19) was declared as a pandemic by the WHO.

The most common symptoms are fever, cough and fatigue. Diarrhea was uncommon [2].

SARS-CoV-2 is thought to be transmitted from person to person mainly through respiratory droplets or close contact [3]. The ocular surface is exposed to the outside environment, which may become a potential gateway for pathogens such as viruses to invade the human body.

Moreover, ACE 2 is a cellular receptor for SARS-CoV- 2 [4]. ACE 2 has also been detected in the human retina [5], vascularised retinal pigment epithelium choroid [6] and cornea and conjunctival epithelia [7].

For these reasons, it’s important to evaluate the clinical spectrum of ocular diseases caused by SARS-CoV- 2 infection.

Moreover, the exponential increase in the number of Covid-19 cases was like a tsunami for health care companies, which were not ready to face this emergency. Ophthalmology departments were also affected by the reorganization of healthcare services. So, it is necessary to rearrange ophthalmologist routine clinical practice in order to control viral spread and try to maximize patient and health-care provider’s safety.

## Main text

Articles were identified by searching PubMed database for articles published between December 2019 and April 2020.

A total of 9 articles was included.

## Result

In a cross-sectional study of Liwen Chen et al., was studied the ocular manifestations and clinical characteristics of 534 cases of COVID-19 in China [8].

The median age of patients was 40 and 50 years at Mobile Cabin Hospital and Tongji Hospital, respectively. There was a similar number of men and women (134/129 and 134/137, respectively) in the two hospitals.

Of 534 COVID-19 patients, 25 patients (4.68%) presented with conjunctival congestion and 3 patients had conjunctival congestion as the initial symptom.

Dry eye (112, 20.97%), blurred vision (68, 12.73%), and foreign body sensation (63, 11.80%) ranked as the top three COVID-19 related ocular symptoms. The authors explain this due to the fact that COVID-19 patients are more likely to have a lot of time to use electronic products.

Only four patients reported a history of eye disease among the 25 cases—two reported conjunctivitis and two reported dry eye. These 25 patients did not report any other eye disease history or any symptoms associated with intraocular diseases (such as iritis, choroiditis, and retinal disease), which suggests that conjunctivitis may be the primary cause of the conjunctival congestion.

Conjunctival congestion and positive RT-PCR in pharyngeal swabs were found at the same time in four COVID-19 patients.

The conclusion of the study is that the conjunctival swab test for SARS-CoV-2 should be performed in patients with conjunctival congestion.

Moreover, among the 25 cases with conjunctival congestion, 18 (72%) had a history of hand-eye contact, 13 with frequent contact and 12 who never washed their hands, suggesting that hand-eye contact is possibly a high risk factor.

A study in the New England Journal of Medicine by Zhong Nanshan and other [1], that analys the clinical features of patients infected with COVID-19, reported nine cases with conjunctival congestion among the 1099 cases enrolled (0.8%).

In a retrospective cohort study by Zohu and others, has been reported 3 cases of conjunctival congestion in 67 COVID-19 patients or suspected cases [9]. Of the cases enrolled in the study, one was positive for the conjunctival sac 2019-nCoV test and two cases were suspicious positive. None of these three patients had ocular symptoms. One patient presented with conjunctivitis as the first symptom but had a negative conjunctival sac 2019-nCoV test. This kind of conjunctivitis has no specific manifestation, and can present in one eye or two eyes. In the early stage, it appears as common conjunctival hyperemia with fewer secretions. It is watery and akin to thin mucus. Occasionally small pieces of conjunctival hemorrhage are seen.

The authors concluded that the incidence of conjunctivitis in patients with new coronavirus pneumonia (NCP) is not high and that the concept that the virus is transmitted through the conjunctival route it is not supported.

Jianhua Xia and collegues evalueted the presence of coronavirus in tears and conjunctival secretions of patients with SARS-CoV-2 infection [10].

Thirty confirmed novel NCP patients were selected at the First Affiliated Hospital of Zhejiang University from 26 January 2020 to 9 February 2020. At an interval of 2 to 3 days, tear and conjunctival secretions were collected twice with disposable sampling swabs for reverse-transcription polymerase chain reaction (RT-PCR) assay.

Only one patient with conjunctivitis found viral RNA in his tear fluid and conjunctival secretion twice. The conjunctivitis was characterized by viral conjunctivitis with conjunctival congestion and aqueous secretion.

No viral RNA was detected in the tear fluid and conjunctival secretions of the severe or common-type patients without conjunctivitis.

The authors concleded that SARS-CoV-2 may be detected in the tears and conjunctival secretions in NCP patients with conjunctivitis. However, the possibility of virus particles in tear and conjunctival secretions in NCP patients without conjunctivitis cannot be completely ruled out.

Marvi Cheema [11] and others reported a case of coronavirus disease 2019 (COVID-19) with an initial medical presentation of keratoconjunctivitis, the first such reported case in North America. The patient, 29 years old, had as first sympton red eye with watery discharge. On examination, she had 20/20 visual acuity OU. Anterior segment examination of the affected eye was remarkable for 2+ conjunctival injection, 3+ follicles, 1 small pseudodendrite in the inferior temporal cornea, and 8 small (0.2 mm) subepithelial infiltrates with overlying epithelial defects at the superior temporal limbus. She was diagnosed with keratoconjunctivitis and a conjunctival swab of the affected eye was positive for the SAR-CoV-2 virus.

Lu Chen and colleagues [12] had describe the ocular manifestations of a hospitalised patient with confirmed 2019 novel coronavirus disease. Thirteen days afer illness onset, the 30 years old reported redness, foreign body sensation and tearing in both eyes without blurred vision. Slit lamp examination identified bilateral moderate conjunctival injection, watery discharge, inferior palpebral conjunctival follicles and tender palpable preauricular lymph nodes. No subconjunctival haemorrhage or pseudomembrane were observed. No lesions on the corneal or anterior chamber inflammation were detected.

He was diagnosed as bilateral acute conjunctivitis and RT-PCR assay demonstrated the presence of viral RNA in conjunctival specimen. The conjunctival swab specimens remained positive for SARS-CoV- 2 on 14 and 17 days after onset. On day 19, RT-PCR result was negative for SARS-CoV- 2.

They concluded that SARS-CoV- 2 is capable of causing ocular complications such as viral conjunctivitis in the middle phase of illness. However, conjunctival sampling might not be useful for early diagnosis because the virus may not appear initially in the conjunctiva.

Zhang et al. [13] found that two patients with conjunctivitis was identified from 72 patients with a laboratory confirmed COVID-19. However, SARS-CoV-2 was found in ocular discharges by RT-PCR only in one COVID-19 patient. Although the incidence of conjunctivitis is extremely low, these results demonstrated that SARS-CoV-2 have shown a capacity to use the eye as a portal of entry and cause ocular disease. The entry of SARS-CoV-2 via the host functional receptor is mediated by ACE2 which is expressed in human cornea and conjunctival tissues.

They concluded that the negative results of conjunctival sac in other 71 patients may be owing to the lower viral concentration, the sampling time lag (mean time 18.15 days), and the lower positive rate of the inefficient diagnostic method.

Ziad and collegue [14] found that tracheal aspirates yelded significantly higher SARS-CoV loads, compared with the nasopharyngeal swab and sputum specimens. This suggests that the viral concentration and genome fraction is diverse in different sites. In consideration of the ocular surface is an open microenvironment, and the viral may transport to the inferior meatus of the nose rapidly, the SARS-CoV-2 concentration in ocular surface is likely to be very low.

De Wit et collegues [15] demonstrated that, in the rhesus macaque model, MERS-CoV RNA could detect in the conjunctiva, and the viral loads could no longer be detected in the conjunctiva 6 days post infection.

Another thing needs to be considered is that the lower positiive rate of RT-PCR makes early of SARS-CoV-2 a challenge. Therefore, improvements in the sensitivity of molecular diagnostic methods need to be taking in the future.

A PubMed search on 24th March 2020 found no evidence that contact lens wearers are more likely to contract COVID-19 than spectacle wearers. The likely belief for this being a concern relates to the fact that SARS-CoV-2 has been isolated in tears, albeit to date, infrequently [16] and also that the virus is known to be transferred by hand contact, and could be transferred to contact lenses during their application and removal. Thus, it is understandable this has been raised as a potential concern for increasing their risk of exposure to the virus. The consistent advice to protect individuals from the virus is to employ frequent handwashing with soap and water. The lipid envelope of the virus can be emulsified by surfactants such as those found in simple soap, which kills the virus [17, 18].Best practice advice for contact lens wearers includes the same instructions that should be impered under all situations, regardless of the COVID-19 pandemic. When using contact lenses, careful and washing with soap ant water followed by hand drying with unused paper towels is paramount. For contact wearers, this should occur before every contact lens application and removal, and such practice reduces the risks of infection and inflammatory responses and is highly effective [19]. It follows that as long as contact lens wearers are using correct hand hygiene techniques, they should be limiting any virus transmission to their ocular surface, and indeed, as already stated, there is currently no evidence that they are at any higher risk of developing COVID-19 infection than non-wearers [20].

To date, there have been no laboratory studies reported on the ability of coronaviruses to adhere to contact lenses, and none on the ability of disinfectants to kill coronaviruses adhered to contact lenses [20].

There is no evidence of the presence of SARS-CoV-2 in the tears or conjunctival tissue of asymptomatic patients and even in those with confirmed disease, the presence of SARS-CoV-2 on the ocular surface is low [16, 21, 22]. Thus, binding of SARS-CoV-2 to contact lenses from the ocular surface in asymptomatic wearers would be unlikely [20].

A recent paper [23] has been cited as suggesting that silicone hydrogels are more likely to bind SARS-CoV-2 than hydrogels. However, this paper did not examine contact lens materials [20]. The inanimate surfaces described in the paper [23] which most closely resemble contact lens materials were “plastic” and silicon rubber. Neither of these materials appropriately represents the complex bulk and surface chemistry of contemporary contact lens materials and cannot be used to reflect the likely binding of any pathogenetic organism to modern day contact lenses [20]. The factors governing the binding of SARS-CoV-2 to inanimate surfaces are so far unknown, but for a variety of waterborne viruses the major driving factors were electrostatic interactions (charge driven), followed by hydrophobic interactions, with only minor impact from van der Waals interactions [20].

## Discussion

The purpose of this review is to highlight the possible ocular presentation of COVID-19 infection and the consequence of the pandemic in the daily ophtalmology routine.

Conjunctivitis (and keratoconjunctivitis) can be the first symptom in infected patients. The virus can be present in tear and conjuntival secretions, requiring maximum attention.

It’s important to understand if COVID-19 could spread through the ocular route or present as the primary infected site.

Ocular implications should also be considered for therapy. In fact, one potential treatment is chloroquine and its derivatives [24, 25], including hydroxychloroquine, which have both antiviral and anti-inflammatory effects. These compounds are effective against SARS-CoV-2 in vitro, but in vivo data are lacking. Although some encouraging outcomes have been reported, further evidence from coordinated multicentre trials is required.

Hydroxychloroquine can induced Retinal Toxicity [26] after long term usage. Retinopathy is rarely seen before 10 or more years of usage at the recommended dosage [27]. However, the doses proposed to treat COVID-19 are 4–5 times higher [28] and this must be considered during the therapy and the ocular implication must be investigated. It’s important to analays the ocular fundus before, during and after the treatment.

The exponential increase in the number of Covid-19 cases was like a tsunami for health care companies, which were not ready to face this emergency. Ophthalmology departments were also affected by the reorganization of healthcare services. We submitted a questionnaire to 14 health care companies present in Piedmont, in Italy.

In all there was a reduction in ambulatory activity, reserved only for urgent non-deferrable services. There was a reduction in shifts in the operating room, reserved for emergencies. In five cases, operating rooms were intended for COVID-19 patients with severe symptoms, sending urgent ophtalmology patients to other better equipped hospitals. Furthermore, in six hospitals, ophthalmology personnel were sent to the COVID-19 departments to carry out non-specialist assistance activities.

A dedicated Covid-free ophthalmology department has been maintained in only four healthcare companies. In other cases, beds with other specialties have been merged, with the aim of freeing up as many hospital wards as possible for patients with COVID-19.

Even when the emergency is over, the guard cannot be lowered. In fact, ophthalmologists and other eye care professionals remain at higher risk of infection, due to the proximity to the patients during the physical examination.

It is important to think carefully about how to take care of patients until the World is declared COVID-free.

**Fig. 1:**
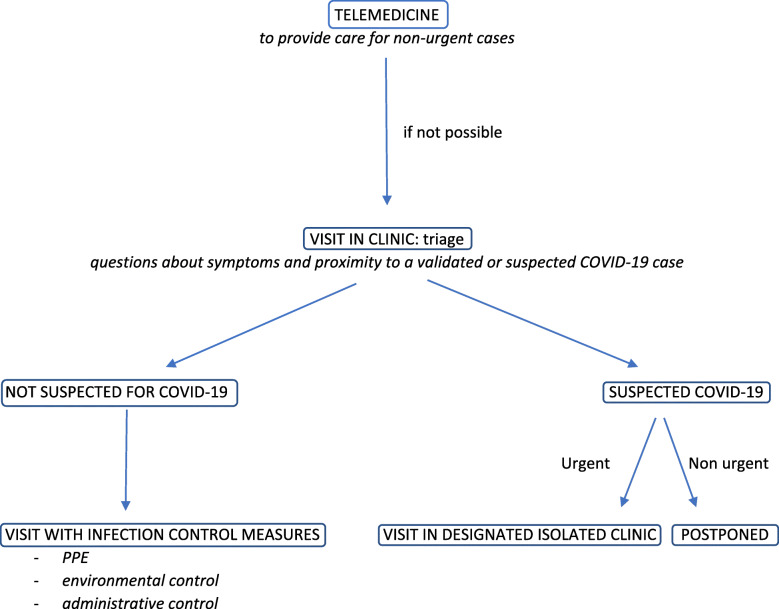
Flowchart to follow in patient management

Several infection control measures are recommended (Fig. 1), subdivided into three categories [29]:

- use of PPE
- environmental control
- administrative control.

In addition, the use of telemedicine must be encouraged to provide care for non-urgent cases. Telemedicine is defined as the use of information technologies to support healthcare between participants who are separated from each other [30]. Fortunately, the current technologies allow an optimal management for less severe cases. “Routine post-op” care for cataract surgery in non-glaucomatous eyes, for example, could likely be cut further via telemedicine.

### PPE

The American Academy of Ophthalmology (AAO) [31] has published a report advising ophthalmologists to wear masks and eye protection when caring for patients potentially infected with COVID-19. In our opinion, it’s important to use of PPE for all cases regardless of SARS status, as well as hand hygiene measures and use of gloves, N95 masks, goggles and gowns. The patient should not speaking during the slit lamp examination.

#### Environmental control

**Fig. 2:**
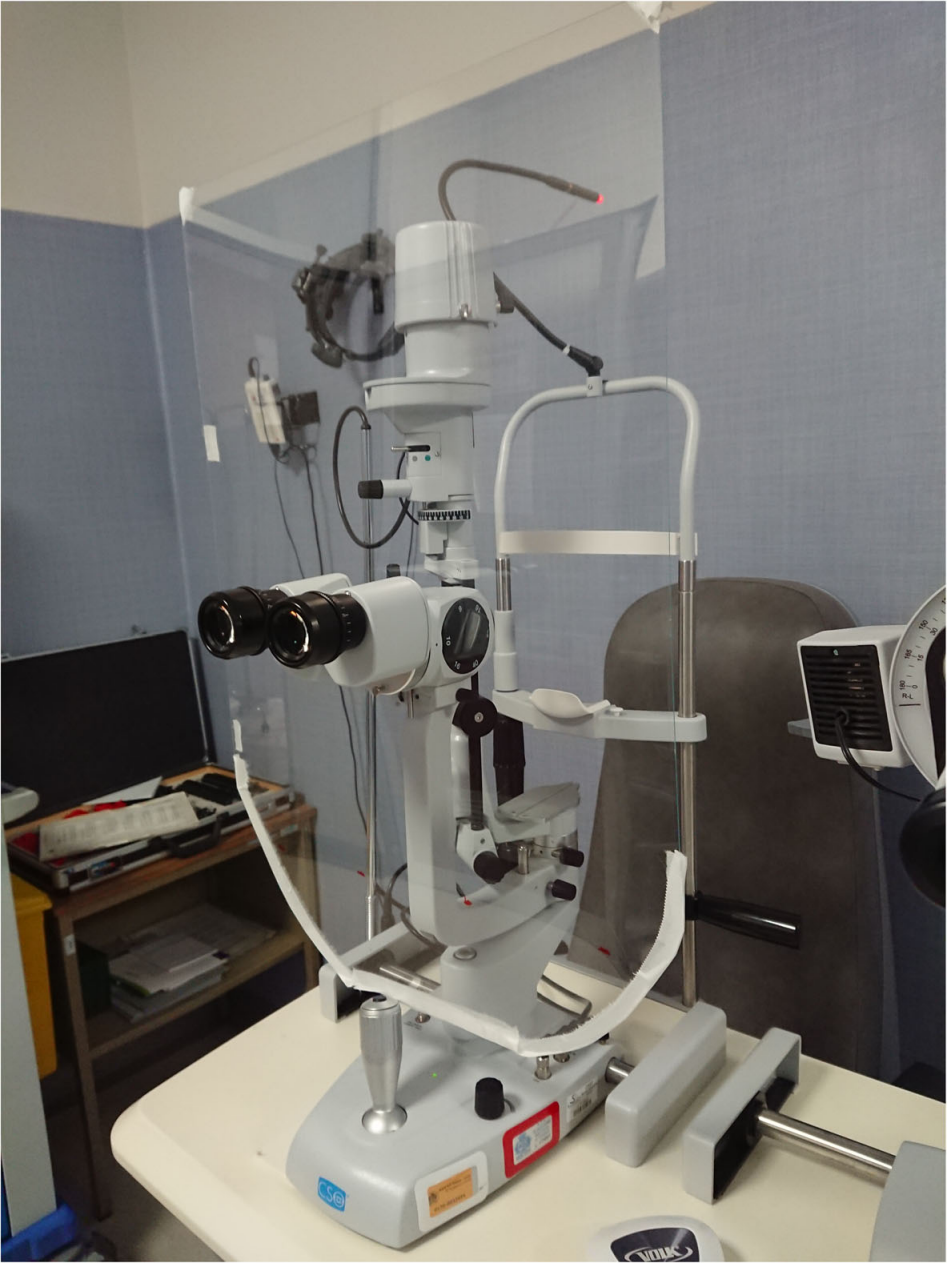
Large plastic protective screens on slit lamps

The installation of large plastic protective screens on slit lamps (Fig. 2) is essential to minimize the risk of droplet contamination between the patient and ophthalmologist. Even AAO [31] recommended this installation.

It’s important the use of disinfectants specific to COVID-19 [32], including diluted household bleach (five tablespoons of bleach per gallon of water) and alcohol solutions with at least 70% alcohol.

Lai et al. [29] reported the importance of air ventilation in waiting areas.

#### Administrative control

Basic survey to identify patients with possible exposure to SARS-CoV- 2 or the development of COVID-19 should be performed.

This should include questions about symptoms (fever, dry cough, sore throat, headache, loss of taste/smell) and proximity to a validated or suspected COVID-19 case as well as travel to an endemic region [31]. This screening should be performed by calling the patient in advance; in alternative, at the front desk once the patient arrives at the clinic.

Balancing between the need to provide care and save sight and the risk of contracting COVID-19 should be considered with respect to each patient.

**Fig. 3:**
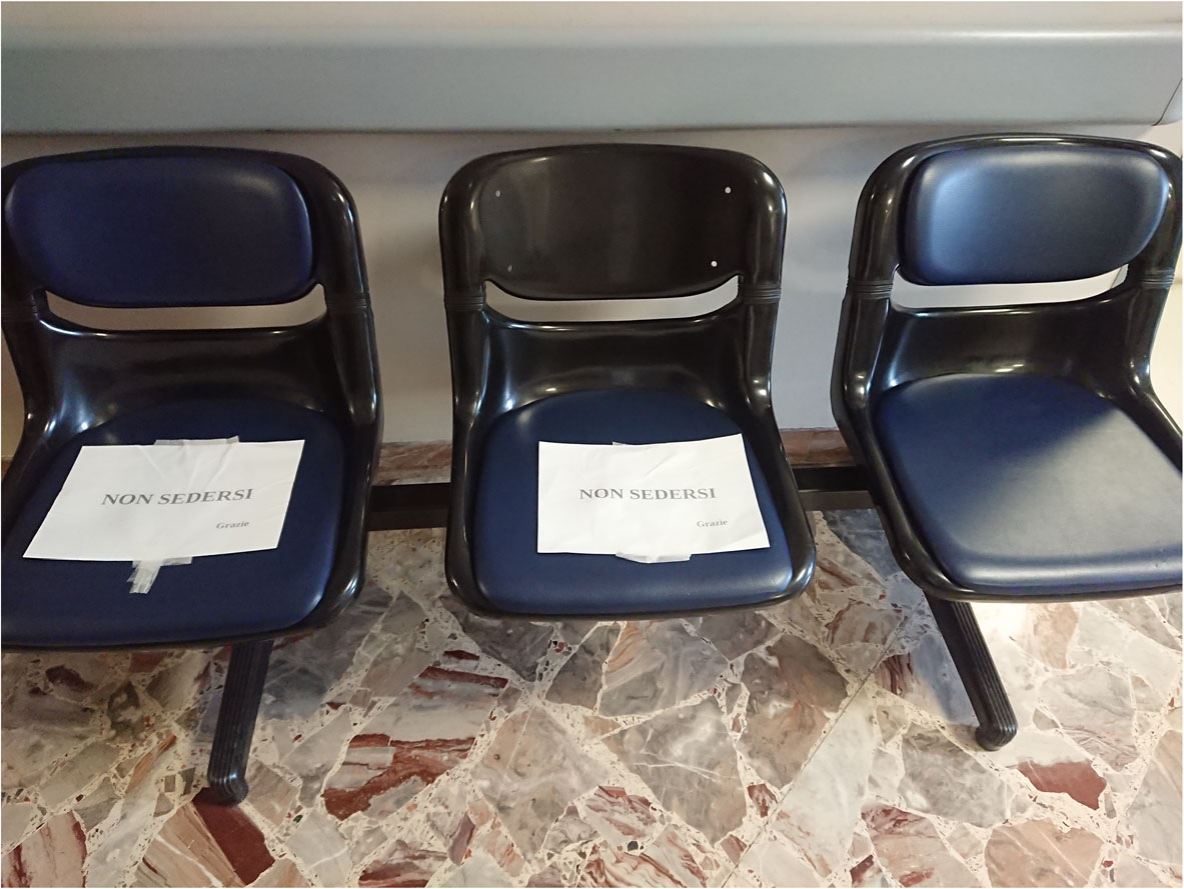
Waiting area with alternating seats

In addition, social distancing must be implemented in waiting areas by blocking alternating seats (Fig. 3).

Further precautions can be taken during the visit [33]:

- the patient should enter without accompanying adults, if possible;
- Imaging devices will necessitate careful cleaning between patients. Automated perimetry needs to be completely revisited, since the perimetry bowl is not only a potential source of viral spread, but also notoriously difficult to clean without damage;
- use of disposable tonometers with single-use protective sleeves. Pneumotonometers and air-puff tonometry, both of which can presumably aerosolize the tear film and viral particles, may need to be avoided;
- use of single-use gonioscopy, laser and hand-held indirect lenses;
- multi-use eye drop bottles (dilating agents, for example) should be limited.

## Conclusion

It is difficult to draw definitive conclusions about the ocular manifestations of COVID-19.

The studies analaysed, in fact, have some limitations. First, the sample size and the covered population consisted mainly of patients with mild disease. Moreover, the studies are often descriptive study, without a correlation analysis. Finally, no normal population was observed in the studies, so a normal control group should be included for comparison in future studies.

Anyway, with the evolving COVID-19 pandemic and with it’s high infectivity, it is necessary to rearrange ophthalmologist routine clinical practice in order to control viral spread and try to maximize patient and health-care provider’s safety.

It is essential to create COVID-free ophthalmology ward and a ward for suspected or confirmed diagnosis of COVID-19. Individuals with non-urgent ophthalmic problems who are suspected, probable or confirmed COVID-19 cases can be postoponed for medical treatment.

If the visit is urgent, they must be evaluated in a designated isolated clinic.

In the long run, it is helpful to capitalize on this experience and reorganize how we offer healthcare to our patients. In this regard, it is useful to consider a Hub and Spoke model, with a greater focus on prevention in the territorial hospital, referring only the most serious cases to major Eye clinics.

## Data Availability

Not applicable.

## Abbreviations

AAO: American Academy of Ophthalmology
ACE 2: Angiotensin-converting enzyme 2
COVID-19: COronaVIrus Disease 19
PPE: Personal Protective Equipment
SARS-CoV-2: Severe Acute Respiratory Syndrome - Coronavirus - 2
WHO: World Health Organization
